# A pathological classification for predicting recurrence and guiding adjuvant therapy in esophageal squamous cell carcinoma following neoadjuvant immunochemotherapy: a two-center cohort study

**DOI:** 10.3389/fonc.2026.1778731

**Published:** 2026-03-13

**Authors:** Jiaming Huang, Hongsheng Xie, Guiqing Zeng, Manhong Yao, Zhifeng Zhang, Zhekai Zhang, Qijun Zheng

**Affiliations:** 1 Jinan University, Guangzhou, Guangdong, China; 2 Department of Thoracic surgery, Jieyang People’s Hospital, Jieyang, Guangdong, China; 3 Department of Cardiovascular Surgery, Shenzhen People’s Hospital (The Second Clinical Medical College, Jinan University, The First Affiliated Hospital, Southern University of Science and Technology), Shenzhen, Guangdong, China

**Keywords:** adjuvant therapy, chemotherapy, esophageal squamous cell carcinoma, neoadjuvant immunotherapy, pathological response, recurrence pattern

## Abstract

**Background:**

Neoadjuvant immunochemotherapy (nICT) has emerged as a promising treatment modality for locally advanced esophageal squamous cell carcinoma (ESCC). However, optimal post-nICT adjuvant strategies remain undefined, and a classification system that integrates both prognosis and recurrence patterns to guide treatment decisions is currently lacking.

**Methods:**

This retrospective study enrolled 283 patients with locally advanced ESCC who underwent nICT with R0 resection between January 2019 and December 2023 at two participating institutions. The primary endpoint was recurrence-free survival (RFS). Secondary endpoints included recurrence patterns, overall survival (OS), locoregional recurrence-free survival (LRFS), and distant metastasis-free survival (DMFS). Survival curves were generated using the Kaplan-Meier method. Propensity score matching was employed for group comparisons and a Cox proportional hazards model was used to identify prognostic factors.

**Results:**

The pathological complete response (pCR) and major pathological response (MPR) rates were 22.6% and 52.3%, respectively. Multivariate analysis identified the tumor regression grade (TRG) and ypN stage as independent predictors of RFS. Both ypN status and TRG were key determinants of recurrence patterns. Based on this, patients were stratified into four subgroups: Group 1 (TRG0-1 ypN0), Group 2 (TRG0-1 ypN+), Group 3 (TRG2-3 ypN0), and Group 4 (TRG2-3 ypN+). This classification demonstrated significant prognostic stratification, with Group 1 having the best prognosis and Group 4 having the worst prognosis. In the entire matched cohort, adjuvant therapy did not significantly improve survival. However, subgroup analyses revealed that adjuvant therapy was associated with a significant improvement in RFS in Group 2 (TRG0-1 ypN+)(HR = 0.16, 95% CI 0.06–0.42, *P*<0.001).

**Conclusion:**

The proposed classification system based on TRG and ypN status effectively stratified the prognosis of patients with ESCC after nICT. This classification enabled the identification of a specific subgroup (TRG0-1 ypN+) that may benefit from postoperative adjuvant treatment.

## Introduction

Esophageal cancer is a highly prevalent gastrointestinal malignancy, ranked eighth in incidence and sixth in mortality among all cancers worldwide (1–3), that poses a significant threat to human health. In China, the incidence and mortality rates of esophageal cancer are among the highest globally, with squamous cell carcinoma accounting for over 90% of cases (4–8). Most patients are diagnosed at a locally advanced stage. The primary treatment for locally advanced ESCC involves neoadjuvant therapy followed by surgery (9–12), with options including neoadjuvant chemotherapy (nCT), neoadjuvant chemoradiotherapy (nCRT), and neoadjuvant immunochemotherapy (nICT). nICT has shown promise in the treatment of locally advanced ESCC (13), and its efficacy and safety have been demonstrated in several clinical trials (14). Although the pCR rate with nICT is lower than that with nCRT, R0 resection rates are comparable (15). Moreover, nICT may offer a superior long-term prognosis compared with nCRT (16–20), making it a suitable option for high-risk patients or those averse to radiotherapy.

However, the optimal adjuvant strategy following nICT and radical esophagectomy remains uncertain (21–25). With diverse options such as radiotherapy, chemotherapy, and immunotherapy, selecting the best postoperative treatment is controversial. Consequently, there is an urgent need for a classification system to aid postoperative prognostic assessment and to guide treatment decisions. Current stratification methods often rely on ypTNM staging combined with surrogate endpoints, such as pathological complete response (pCR) and major pathological response (MPR) (26–29). However, the prognostic discriminatory power of ypTNM can be diminished by the “downstaging” effect of neoadjuvant therapy. pCR and MPR are unidimensional metrics and thus fail to fully capture the multidimensional risk of recurrence. These methods are suboptimal for predicting postoperative recurrence and are insufficient for guiding treatment.

**Figure 1:**
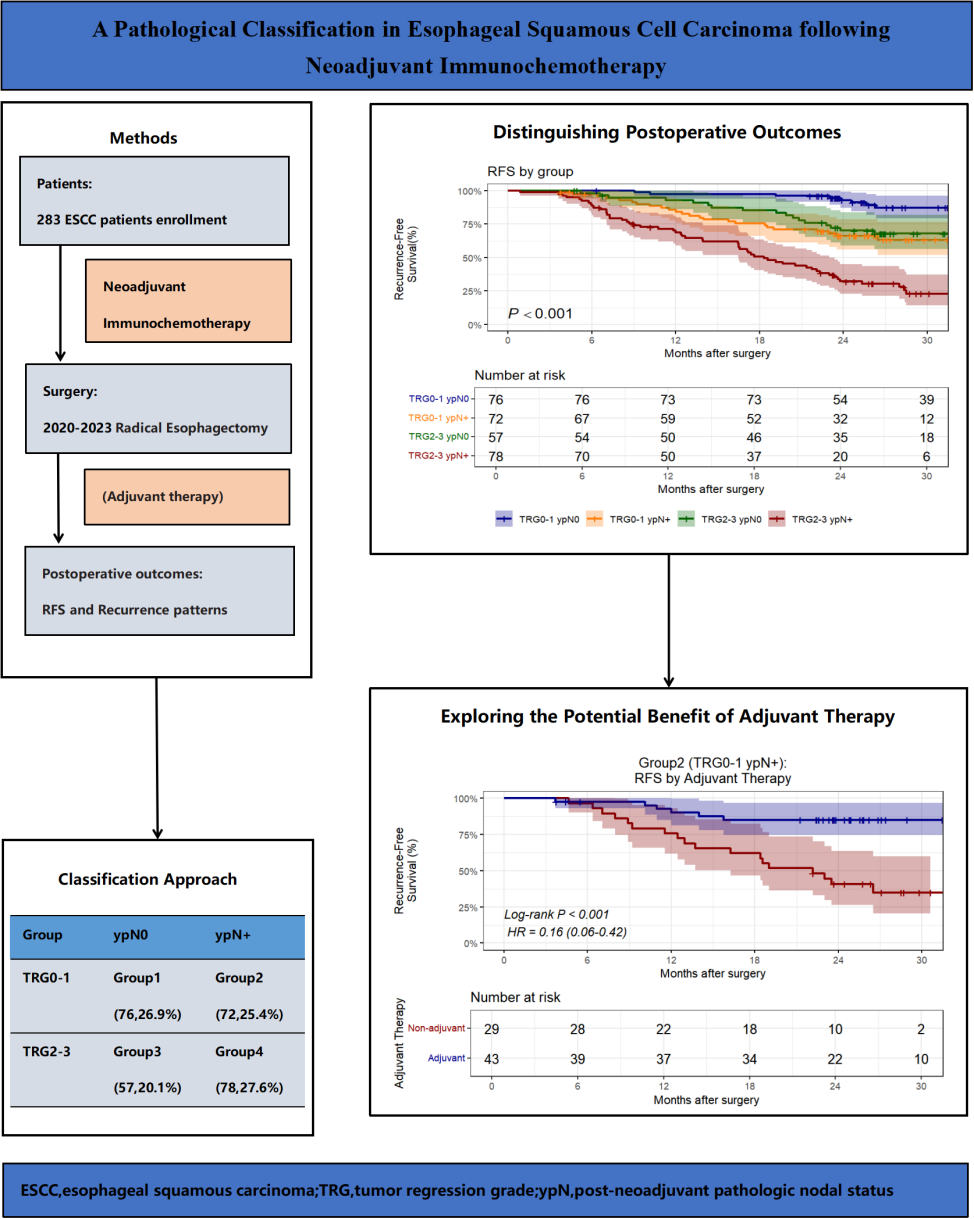
Overview of the study design, key findings, and implications.

To address these limitations, we developed a novel prognostic stratification model based on TRG and ypN status and explored the potential benefits of adjuvant therapy by analyzing postoperative pathological data and outcomes (**Figure 1**).

## Methods

The study was approved by the Ethics Committees of Jieyang People’s Hospital (No.2025062) and Shenzhen People’s Hospital and was conducted in accordance with the principles of the Declaration of Helsinki. The requirement for informed consent was waived due to the retrospective nature of the study.

### Study design and patient population

This retrospective study included patients with locally advanced ESCC (cT1-2N1-3 M0 or cT3-4aN0-3 M0, according to criteria from the AJCC, 8th edition) who underwent R0 resection after receiving nICT at Jieyang People’s Hospital or Shenzhen People’s Hospital between January 2019 and December 2023.

Exclusion criteria were: a) history of other malignancies within 5 years; b) incomplete clinical or pathological data; c) non-squamous cell carcinoma or mixed histology; d) neoadjuvant therapy not involving chemotherapy combined with immune checkpoint inhibitors e) non-R0 resection; and f) death due to surgical complications or other causes within a 3-month perioperative period.

### Study endpoints

The primary endpoint was recurrence-free survival (RFS), defined as the time from surgery to confirmed or highly suspected recurrence or death from any cause. The secondary endpoints included recurrence pattern, overall survival (OS), locoregional recurrence-free survival (LRFS), and distant metastasis-free survival (DMFS). All survival times were calculated starting from the date of surgery.

pCR was defined as ypT0N0M0 or ypTisN0M0, and MPR was defined as ≤10% residual tumor in the primary lesion, according to AJCC 8th edition criteria. Tumor regression grading (TRG) was assessed using AJCC 8th edition criteria: TRG0 (no residual tumor), TRG1 (single cells or rare small groups of cancer cells), TRG2 (more than single cells or rare small groups of cancer cells with evident tumor regression), and TRG3 (extensive residual tumor or no regression) (30, 31).

### Treatment and follow-up

All tumors were deemed resectable or potentially resectable prior to treatment and patients received 2–4 cycles of nICT consisting of a taxane-platinum doublet combined with a PD-1/PD-L1 inhibitor. Following neoadjuvant therapy, 95.3% of the patients underwent McKeown esophagectomy and 25.6% underwent three-field lymphadenectomy. R0 resection was achieved in all surgeries, and postoperative radiotherapy was not routinely administered. Specific adjuvant treatment regimens were determined through multidisciplinary team discussions, considering the tumor stage (ypT, ypN), pCR/MPR status, patient age, performance status (PS) scores, and patient preferences. Adjuvant therapy was initiated within 1 to 2 months of surgery. In most cases, 2-4 cycles postoperative cycles of adjuvant chemotherapy were administered and adjuvant immunotherapy was maintained for 1 year. Patients of the two centers were followed up every three months for the first two years post-surgery and every six months thereafter. Follow-up examinations included enhanced CT of the neck, chest, and upper abdomen; tumor marker assays; and gastroscopy or PET-CT when indicated. No patients who were missing follow-ups/attritions were included in this study.

### Definitions and location of recurrence

Recurrence patterns were categorized as locoregional recurrence (LR), distant metastasis (DM), or synchronous recurrence (LR+DM); the latter was defined as recurrences occurring within one month of each other. According to the AJCC 8th edition criteria, LR refers to recurrence at the anastomosis, residual esophagus, gastric conduit, or regional lymph nodes diagnosed via gastroscopy, PET-CT, enhanced CT, or biopsy. DM refers to lymph node or organ involvement outside the locoregional area, as confirmed by PET-CT, enhanced CT, or biopsy.

### Statistical analysis

Categorical variables are presented as counts and percentages; continuous variables are expressed as mean ± standard deviation if normally distributed, or median and interquartile range otherwise. The Kaplan-Meier method was used to estimate the RFS and OS. Survival curves were compared using the log-rank test and pairwise comparisons were adjusted using the Bonferroni method. All analyses were performed using R software (version 4.4.3). Statistical significance was defined as a two-sided *P* value of < 0.05.

To reduce confounding in the assessment of adjuvant therapy, propensity score matching (PSM) was employed. The covariates included age, ypT stage, ypN stage, and TRG, which were selected *a priori* based on clinical relevance. A 1:1 nearest-neighbor matching algorithm with a caliper width of 0.2 was used without replacement. Covariate balance before and after matching was assessed using standardized mean differences.

Univariate and multivariate Cox proportional hazards models were used to identify factors associated with postoperative RFS in the entire cohort. Variables with *P* < 0.05 in univariate analysis, along with clinically relevant variables (e.g. adjuvant therapy), were included in the multivariate model. Stratification into risk groups was based on key prognostic factors identified by multivariate Cox modeling, considering clinical relevance.

## Results

### Patient characteristics and outcomes

**Figure 2:**
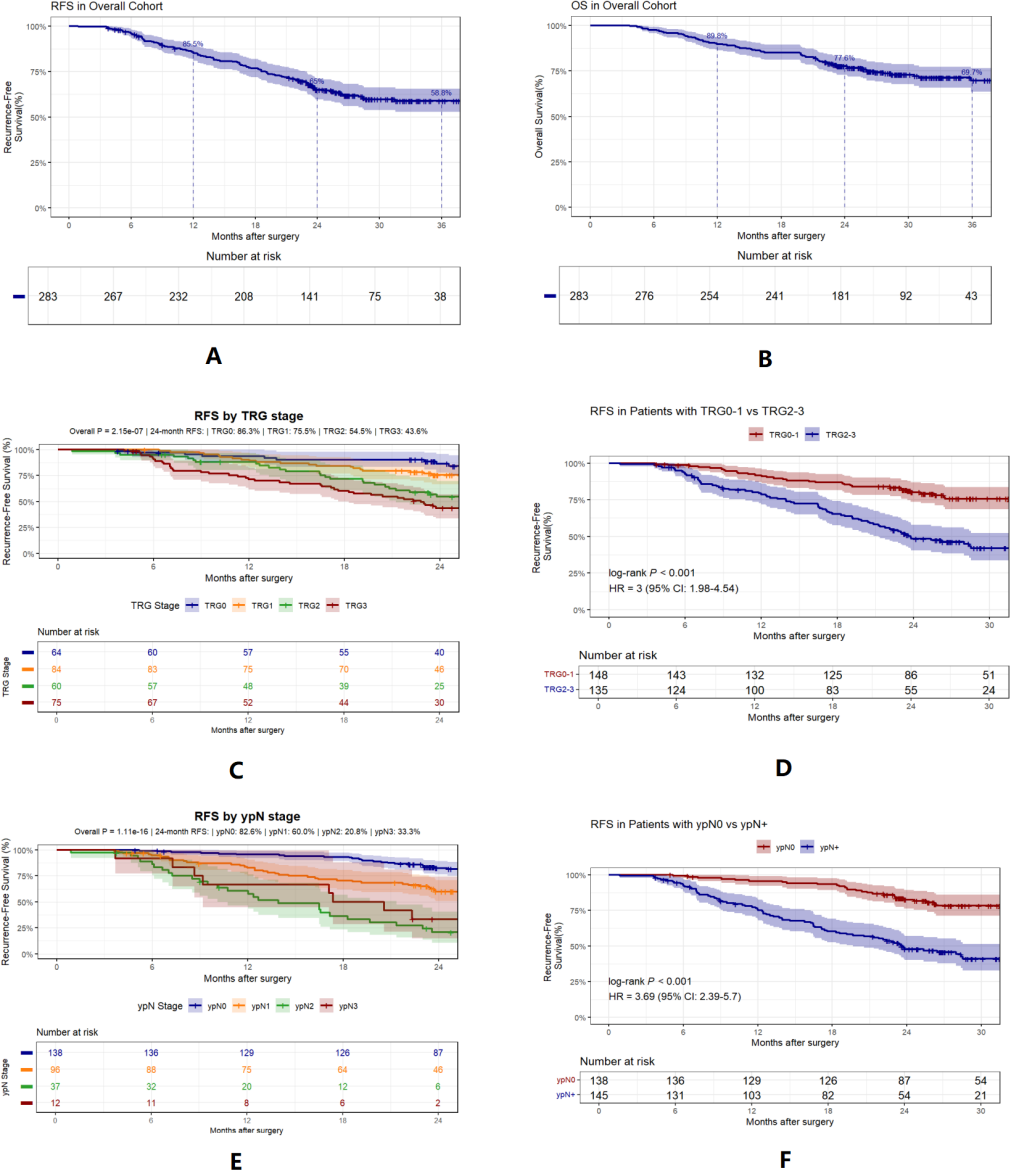
Kaplan-Meier curves for survival outcomes. **(A, B)**, RFS and OS in the overall cohort. **(C, D)**, RFS stratified by tumor regression grade(TRG). **(E, F)**, RFS stratified by post-neoadjuvant pathologic nodal status(ypN).

In total, 283 patients were enrolled between January 2019 and December 2023 (**Supplementary Figure A1**). **Table 1** summarizes baseline characteristics and treatments. The pCR and MPR rates for the entire cohort were 22.6% and 52.3%, respectively. **Figure 2** shows RFS and OS curves for the total population.

**Table 1:** Baseline characteristics.

Characteristic	N = 283
Sex, n (%)	
Female	87 (30.7%)
Male	196 (69.3%)
**Age, y, mean ± SD**	67.2 ± 7.2
**Weight, kg, mean ± SD**	55.9 ± 10.1
**Height, cm, mean ± SD**	162.4 ± 7.4
**BMI, kg/m², mean ± SD**	21.1 ± 3.3
**Location, n (%)**	
Upper thoracic	31 (11.0%)
Middle thoracic	153 (54.1%)
Lower thoracic	99 (35.0%)
**Tumor length, cm, mean ± SD**	4.8 ± 1.9
**Neoadjuvant Immunotherapy, n (%)**	
Karelizumab	138 (48.8%)
Tirelizumab	39 (13.8%)
Srolizumab	31 (11.0%)
Sindilizumab	30 (10.6%)
Teraplizumab	23 (8.1%)
Navulizumab	22 (7.8%)
Neoadjuvant Chemotherapy, n (%)	
Paclitaxel* + Carboplatin	156 (55.1%)
Paclitaxel* + Cisplatin	109 (38.5%)
Other	18 (6.4%)
**Neoadjuvant cycles, n (%)**	
2	175 (61.8%)
3-4	108 (38.2%)
ypT stage, n (%)	
0	91 (32.2%)
1	25 (8.8%)
2	44 (15.5%)
3	117 (41.3%)
4a	6 (2.1%)
ypN stage, n (%)	
0	133 (47.0%)
1	96 (33.9%)
2	40 (14.1%)
3	14 (4.9%)
Tumor regression grade, n (%)	
0	64 (22.6%)
1	84 (29.7%)
2	60 (21.2%)
3	75 (26.5%)
**Lymph node numbers, n, mean ± SD**	25.5 ± 11.0
**Pathological complete response, n (%)**	64 (22.6%)
**Major pathological response, n (%)**	148 (52.3%)
**Neural invasion, n (%)**	112 (39.6%)
**Vascular tumor thrombus, n (%)**	75 (26.5%)
PD-L1 expression, n (%)	
<1%	124 (43.8%)
≥1%	159 (56.2%)
Ki-67 index, n (%)	
<30%	100 (35.3%)
30%-60%	112 (39.6%)
>60%	71 (25.1%)
Recurrence pattern, n (%)	
No recurrence	179 (63.3%)
LR	28 (9.9%)
DM	42 (14.8%)
LR+DM	34 (12.0%)
Adjuvant therapy, n (%)	
**NO**	103 (36.4%)
**Postoperative adjuvant immunochemotherapy**	131(46.3%)
**Postoperative adjuvant immunotherapy**	49 (17.3%)

SD, Standard deviation, *Paclitaxel includes standard, albumin-bound, or liposomal forms.

Bold text indicates key baseline characteristics of the study population.

After PSM, 68 matched pairs were included in the overall assessment of the efficacy of adjuvant therapy. Covariates were well-balanced post-matching, as indicated by the standardized mean differences (**Supplementary Figures A2, A3**). The baseline characteristics were generally comparable between the matched cohorts (**Supplementary Table A1**). In the matched cohorts, no statistically significant difference in RFS was observed between the patients who received adjuvant therapy and those who did not (**Supplementary Figures A4, A5**).

### Factors affecting postoperative recurrence

**Figure 3:**
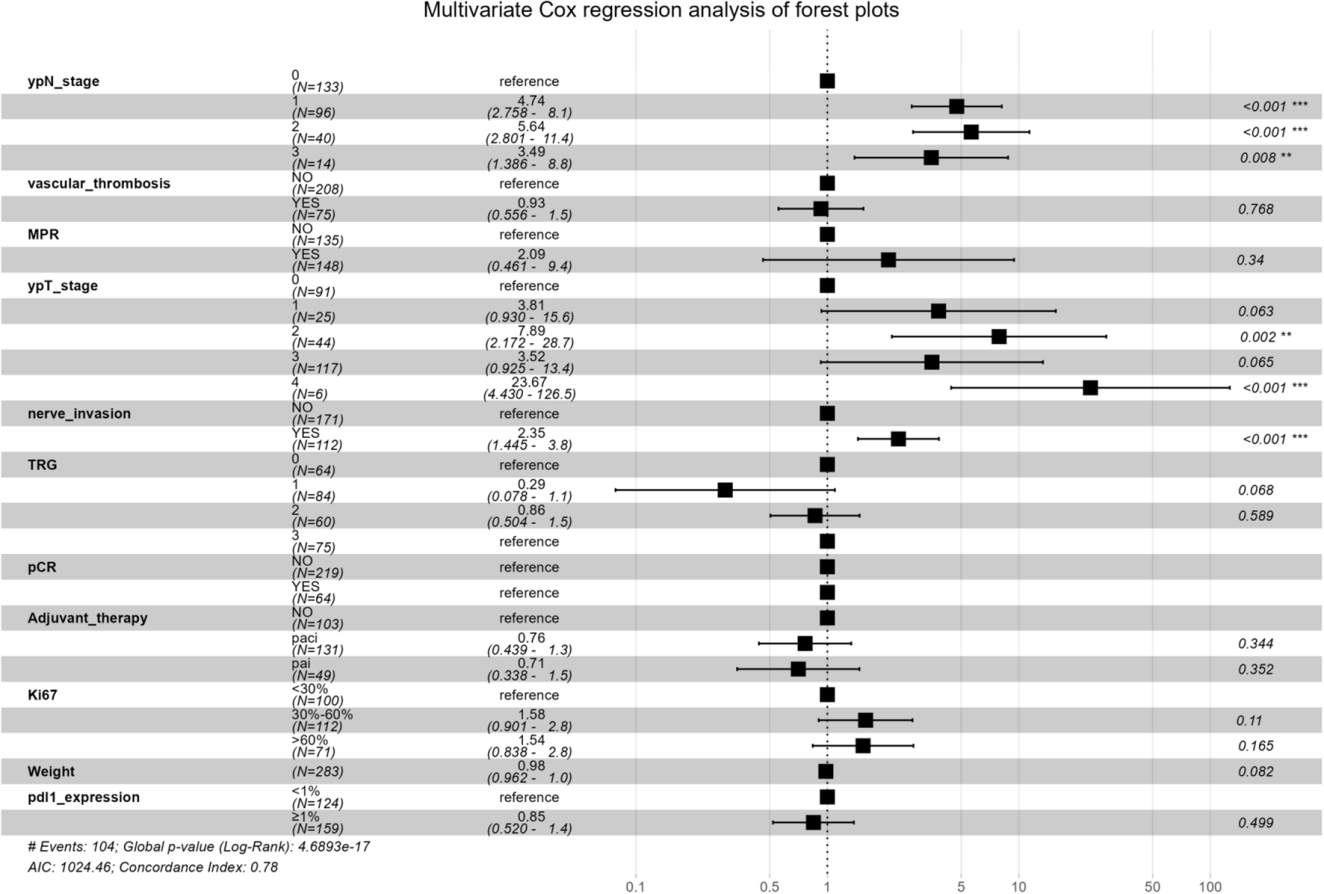
HR > 1 indicates that the factor is a risk factor for increased recurrence risk, HR < 1 indicates a protective factor. The dashed line (HR=1) is the reference line for no effect. MPR, major pathologic response; TRG, tumor regression grade; pCR, pathologic complete response.

Univariate Cox regression analysis identified weight, TRG, pCR, MPR, ypT status, ypN status, nerve invasion, vascular thrombosis, *pdl1* expression and Ki-67 as significant variables (**Supplementary Figure A6**, **Supplementary Table A2**). Multivariate analysis confirmed TRG and ypN stage as independent factors affecting RFS (**Figure 3**, **Supplementary Table A3**).

### Recurrence patterns and influencing factors

**Figure 4:**
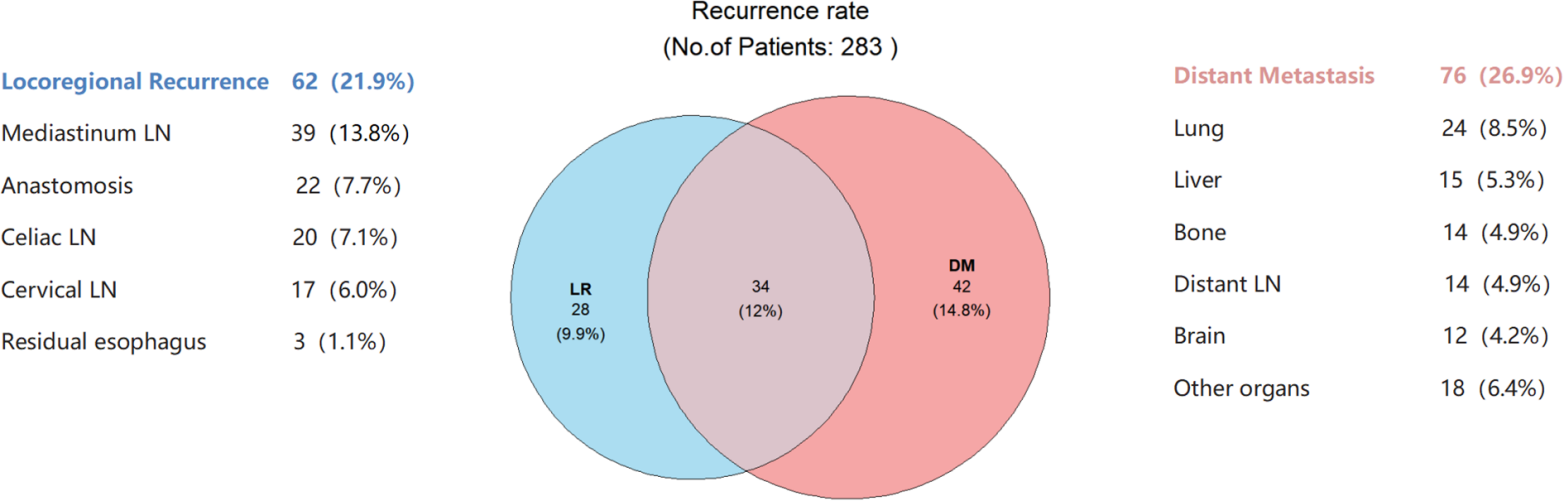
Distribution of recurrence sites. The blue and light red circles represent patients with LR and DM, respectively; overlaps indicate synchronous recurrence. Side annotations show recurrence sites and case counts, with percentages indicating their proportion of the total cohort. LR, Locoregional recurrence; DM, distant metastasis; LN, lymph nodes.

The median follow-up was 25.8 months (range 5.8–56.3 months). Recurrence occurred in 104 of the 283 patients (36.7%), with distant metastasis being the most common pattern (**Figure 4**).

### Classification methods and postoperative outcomes

Further analysis showed that TRG status was highly correlated with RFS. For ease of analysis, TRG was divided into TRG0-1 and TRG2-3 (**Figures 2C, D**). Further analysis revealed that patients with ypN1, ypN2, or ypN3 stage disease had significantly worse RFS than those with ypN0 stage disease. However, the differences between ypN1 and ypN3 stages were not significant (**Figures 2E, F**). Consequently, ypN status was dichotomized into ypN0 and ypN+ for subsequent analyses.

**Figure 5:**
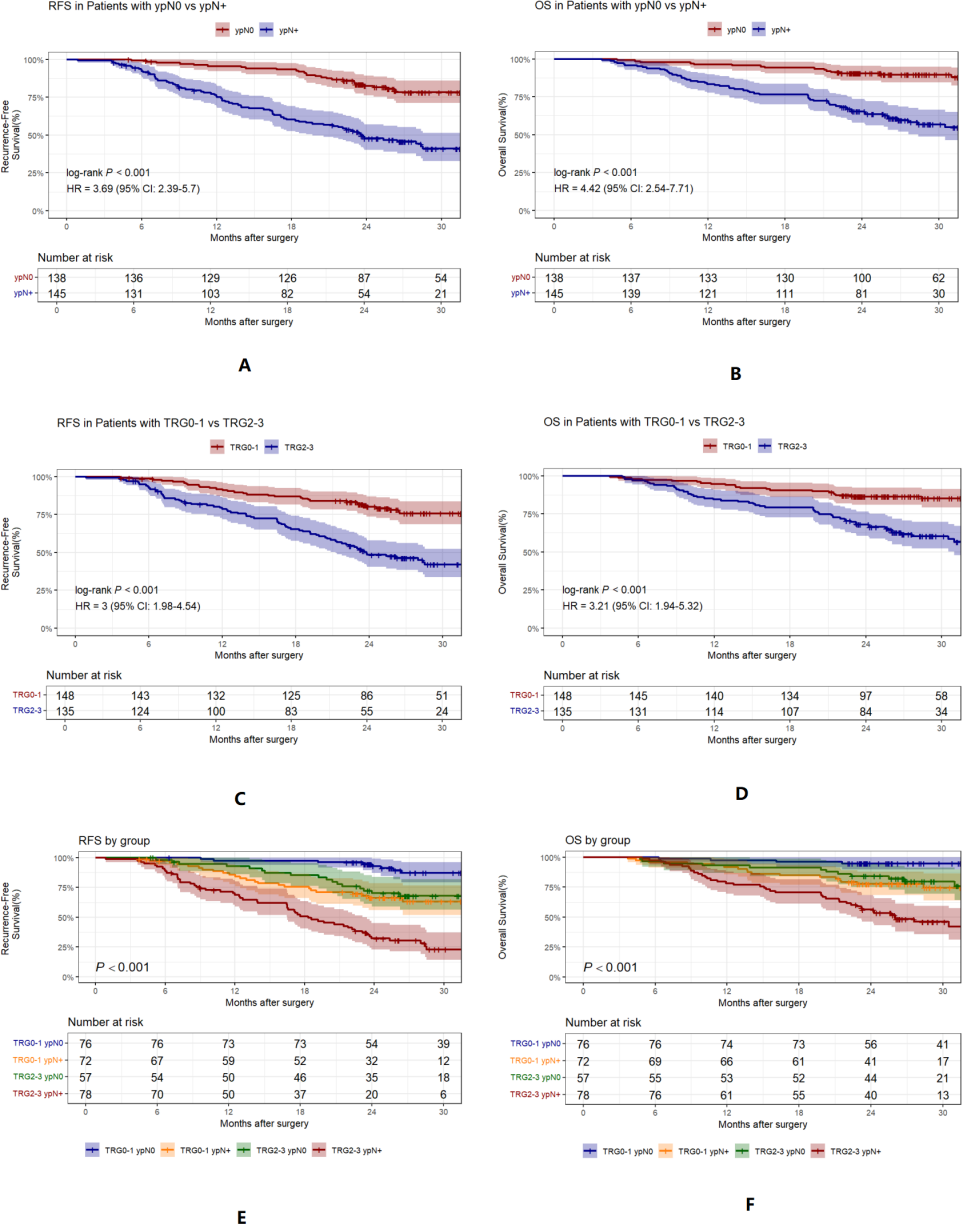
Kaplan-Meier curves for RFS and OS, stratified by TRG, ypN, and group. **(A, B)**, RFS and OS by ypN0 vs ypN+. **(C, D)**, RFS and OS by TRG0-1 vs TRG2-3. **(E, F)**, RFS and OS by group. TRG, tumor regression grade.

Based on these findings and the clinical rationale, we proposed a classification method incorporating TRG and ypN status, dividing the patients into four subgroups: Group1 (TRG0-1 ypN0; n=76; 26.9%), Group 2 (TRG0-1 ypN+; n=72; 25.4%), Group 3 (TRG2-3 ypN0; n=57; 20.1%), and Group 4 (TRG2-3 ypN+; n=78; 27.6%) (**Table 2**). **Figure 5** illustrates survival stratified by ypN status and TRG (A-D), and by the combined groups (E-F). Group 1 had the best prognosis, while Group 4 had the worst, and Groups 2 and 3 exhibited intermediate prognoses.

**Table 2:** Recurrence patterns stratified by group.

Pattern	Group1(TRG0-1 ypN0)(N=76)	Group2(TRG0-1 ypN+)(N=72)	Group3(TRG2-3 ypN0)(N=57)	Group4(TRG2-3 ypN+)(N=78)	*P*
Recurrence	8 (10.5)	25 (34.7)	17 (29.8)	54 (69.2)	< 0.001
LR	1 (1.3)	9 (12.5)	4 (7.0)	14 (17.9)	0.005
DM	4 (5.3)	12 (16.7)	7 (12.3)	19 (24.4)	0.009
LR+DM	3 (3.9)	4 (5.6)	6 (10.5)	21 (26.9)	< 0.001

### Efficacy of adjuvant therapy by group

**Figure 6:**
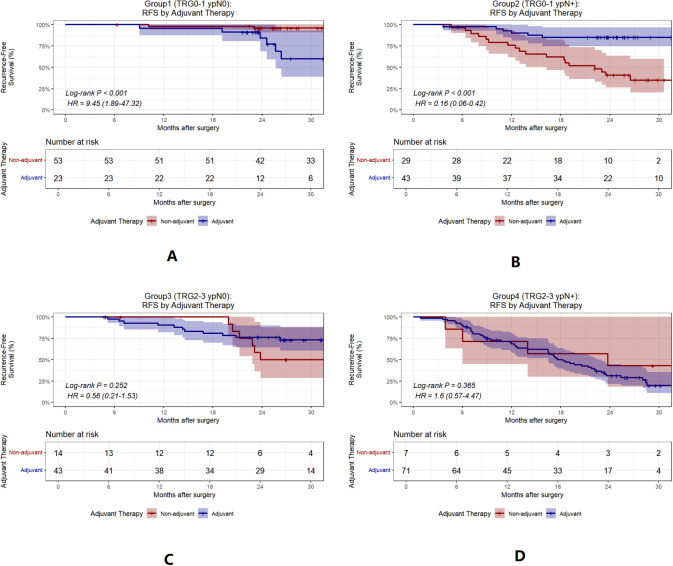
Kaplan-Meier curves for RFS by adjuvant therapy in each group. **(A–D)**, RFS by adjuvant vs non-adjuvant therapy in groups 1 to 4. Non-adjuvant, No adjuvant therapy; adjuvant, postoperative adjuvant immunochemotherapy or postoperative adjuvant immunotherapy. TRG, tumor regression grade.

**Figure 7:**
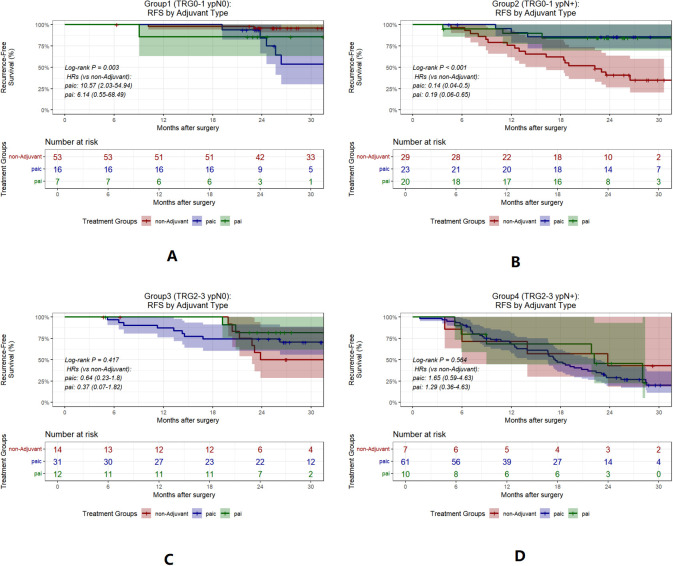
Kaplan-Meier curves for RFS by adjuvant type in each group. **(A–D)**, RFS by paic, pai vs non-adjuvant therapy in groups 1 to 4. Non-adjuvant, No adjuvant therapy; paic, postoperative adjuvant immunochemotherapy; pai, postoperative adjuvant immunotherapy. TRG, tumor regression grade.

To evaluate how this classification informs adjuvant therapy, we reanalyzed treatment outcomes by subgroup. In Group 2, adjuvant therapy was associated with significantly improved RFS (HR = 0.16, 95% CI 0.06–0.42, *P*<0.001)(**Figure 6**). We further analyzed the differences in postoperative adjuvant treatment modalities and found that both postoperative adjuvant immunochemotherapy and postoperative adjuvant immunotherapy benefited group 2 (TRG0-1 ypN+) (**Figure 7**).

## Discussion

nICT has transformed the management of locally advanced ESCC; however, the postoperative recurrence risk and adjuvant strategies remain unclear. This study developed and validated a simple binary classification system based on TRG and ypN status to aid postoperative prognostic assessment and guide adjuvant therapy. Lin et al. (32) also reported the use of MPR and ypN status to predict recurrence risk after nICT. Compared to MPR, TRG offers a more detailed reflection of the tumor response to nICT. Biologically, TRG and lymph node responses are correlated with distinct processes, both of which are associated with a poor prognosis. This combination provides a more comprehensive assessment of the efficacy of neoadjuvant therapy efficacy (31). In our propensity-matched cohort, adjuvant therapy did not confer a survival benefit on the overall population, suggesting limitations of the current empiric adjuvant regimens. However, as this was a retrospective study where adjuvant therapy was primarily administered to patients with high-risk features, the observed benefits could be limited, and the results should be interpreted cautiously.

Current post-nICT decisions rely heavily on ypTNM staging and surrogate endpoints, such as pCR/MPR (28, 29, 33). However, the prognostic power of ypTNM is often attenuated by neoadjuvant “downstaging”, and although the unidimensional metrics pCR and MPR (34–36)identify favorable responders, they are insufficient for comprehensively assessing recurrence risk (24, 37, 38). Our study confirmed that TRG and ypN status are mutually independent prognostic predictors. TRG can reflect the effect of primary tumor lesions, and the ypN status can reflect changes in lymph nodes after nICT. TRG 0-1 corresponds to an MCR, indicating high treatment efficacy, whereas TRG 2-3 indicates poor or no pathological response. A combination of these factors revealed profound tumor heterogeneity. Group 1 (TRG0-1 ypN0) represented a “favorable response” cohort, sensitive to systemic therapy with no nodal residue; their excellent prognosis suggests that a “watch-and-wait” approach may be most appropriate, avoiding overtreatment. Group 4 (TRG2-3 ypN+) constituted a “high-risk subgroup”, combining primary resistance with metastatic potential, clearly defining an ultra-high-risk group warranting exploration of intensive adjuvant therapies (e.g., novel ADC agents, combined radiotherapy). In Group 2 (TRG0-1 ypN+), adjuvant therapy significantly improved RFS (HR = 0.16, 95% CI 0.06–0.42, *P*<0.001), indicating that this subgroup derived substantial benefit, potentially extending the gains of immunotherapy. Group 3 (TRG2-3 ypN0) had an intermediate prognosis and did not benefit from postoperative adjuvant therapy. Recurrence risks in this group might stem from localized micrometastases or hematogenous spread; tailoring adjuvant strategies (e.g. targeted radiotherapy to the primary site or modified systemic therapy) could be beneficial, but requires validation in larger studies.

Our data further confirm that the predominant failure pattern after nICT is distant metastasis rather than local recurrence. This contrasts with the higher local recurrence rates after nCRT and emphasizes that the core objective of any post-nICT adjuvant strategy should be to intensify systemic control. Our classification model, particularly the efficacy of adjuvant therapy in Group 2, likely reflects the successful systemic eradication of micrometastases, thereby reducing distant recurrence.

The limitations of this study include its retrospective design, limited sample size (particularly in certain subgroups), and the inability to assess postoperative radiotherapy, as it was not routinely performed. Furthermore, the median follow-up duration was only 25.8 months, which is relatively short for a definitive oncological prognosis. These limitations necessitate cautious interpretation of the results. Future prospective multicentre studies are warranted to validate this classification model.

In summary, the TRG-ypN binary classification system effectively differentiated the prognosis of patients with ESCC after nICT and identified subgroups likely to benefit from adjuvant therapy. Although its clinical utility requires further prospective validation, this study offers a framework for personalized postoperative management.

## Conclusion

Our classification system based on TRG and ypN status effectively stratified the prognosis of patients with ESCC following nICT and identified specific populations, such as TRG0-1 ypN+ patients, who may benefit from postoperative adjuvant therapy, thereby providing a valuable basis for individualized treatment decisions.

## Data Availability

The original contributions presented in the study are included in the article/supplementary material. Further inquiries can be directed to the corresponding author/s.

## References

[1] Wu Y He S Cao M Teng Y Li Q Tan N . Comparative analysis of cancer statistics in China and the United States in 2024. Chin Med J (Engl). (2024) 137:3093–100. doi: 10.1097/CM9.0000000000003442, PMID: 39654104 PMC11706596

[2] Wilson BE Wright K Sengar M Sullivan R Pearson SA Barton MB . Analysis of 2023 World Health Organization cancer Essential Medicines List and concordance with resource-stratified guidelines. J Natl Cancer Inst. (2025) 117:2010–20. doi: 10.1093/jnci/djaf100, PMID: 40408185 PMC12505124

[3] Qi L Sun M Liu W Zhang X Yu Y Tian Z . Global esophageal cancer epidemiology in 2022 and predictions for 2050: A comprehensive analysis and projections based on GLOBOCAN data. Chin Med J (Engl). (2024) 137:3108–16. doi: 10.1097/CM9.0000000000003420, PMID: 39668405 PMC11706580

[4] Diao X Guo C Jin Y Li B Gao X Du X . Cancer situation in China: an analysis based on the global epidemiological data released in 2024. Cancer Commun (Lond). (2025) 45:178–97. doi: 10.1002/cac2.12627, PMID: 39659114 PMC11833671

[5] Collatuzzo G Abe SK Rahman MS Parvin R Inoue M Boffetta P . Estimating the burden of cancer attributable to tobacco in Bangladesh in 2020. J Cancer Policy. (2025) 45:100614. doi: 10.1016/j.jcpo.2025.100614, PMID: 40619062

[6] Al-zahrani SA Morgan E Zahwe M Fouad H Bray F . Burden of five major types of gastrointestinal cancer in the Eastern Mediterranean Region. BMJ Open Gastroenterol. (2025) 12. doi: 10.1136/bmjgast-2024-001577, PMID: 39971587 PMC11840892

[7] Zhou J Sun K Wang S Chen R Li M Gu J . Associations between cancer family history and esophageal cancer and precancerous lesions in high-risk areas of China. Chin Med J (Engl). (2022) 135:813–9. doi: 10.1097/CM9.0000000000001939, PMID: 35026773 PMC9276202

[8] Yang CS Chen XL . Research on esophageal cancer: With personal perspectives from studies in China and Kenya. Int J Cancer. (2021) 149:264–76. doi: 10.1002/ijc.33421, PMID: 33270917 PMC8141013

[9] Zhang Y Xu W Wu M Li Y Chen G Cheng Y . Survival risk stratification based on prognosis nomogram to identify patients with esophageal squamous cell carcinoma who may benefit from postoperative adjuvant therapy. BMC Cancer. (2024) 24:1330. doi: 10.1186/s12885-024-13085-w, PMID: 39472872 PMC11520824

[10] Zhang Q Zhang T Gu J Zhang X Mao Y Zhu Y . Survival benefits of postoperative radiotherapy in esophageal cancer during the immunotherapy era:a retrospective cohort study based on the SEER database and a single-center registry in China. Front Immunol. (2025) 16:1548520. doi: 10.3389/fimmu.2025.1548520, PMID: 40066458 PMC11891367

[11] Yasuda T Nishiki K Hiraki Y Kato H Iwama M Shiraishi O . Phase II adjuvant cancer-specific vaccine therapy for esophageal cancer patients curatively resected after preoperative therapy with pathologically positive nodes; possible significance of tumor immune microenvironment in its clinical effects. Ann Surg. (2022) 275:e155–e62. doi: 10.1097/SLA.0000000000003880, PMID: 33055588

[12] Yan Y Feng X Li C Lerut T Li H . Treatments for resectable esophageal cancer: from traditional systemic therapy to immunotherapy. Chin Med J (Engl). (2022) 135:2143–56. doi: 10.1097/CM9.0000000000002371, PMID: 36525602 PMC9771193

[13] Fang M Chen M Du X Chen S . Predictive nomogram for postoperative atrial fibrillation in locally advanced esophageal squamous carcinoma cell with neoadjuvant treatment. Front Surg. (2022) 9:1089930. doi: 10.3389/fsurg.2022.1089930, PMID: 36684273 PMC9845906

[14] Xu J Cai Y Hong Z Duan H Ke S . Comparison of efficacy and safety between neoadjuvant chemotherapy and neoadjuvant immune checkpoint inhibitors combined with chemotherapy for locally advanced esophageal squamous cell carcinoma: a systematic review and meta-analysis. Int J Surg. (2024) 110:490–506. doi: 10.1097/JS9.0000000000000816, PMID: 37800587 PMC10793745

[15] Ge Q Guo C Ma Y Li J Lian J Lu T . Comparative efficacy and safety of neoadjuvant immunochemotherapy versus chemotherapy in locally advanced oesophageal squamous cell carcinoma: A dual-centre retrospective study. Eur J Cardiothorac Surg. (2025) 67. doi: 10.1093/ejcts/ezaf268, PMID: 40754841

[16] Xie SH Yang LT Zhang H Tang ZL Lin ZW Chen Y . Adjuvant therapy provides no additional recurrence-free benefit for esophageal squamous cell carcinoma patients after neoadjuvant chemoimmunotherapy and surgery: a multi-center propensity score match study. Front Immunol. (2024) 15:1332492. doi: 10.3389/fimmu.2024.1332492, PMID: 38375480 PMC10875462

[17] Xiao X Hong HG Zeng X Yang YS Luan SY Li Y . The efficacy of neoadjuvant versus adjuvant therapy for resectable esophageal cancer patients: A systematic review and meta-analysis. World J Surg. (2020) 44:4161–74. doi: 10.1007/s00268-020-05721-w, PMID: 32761259

[18] Wang Z Shao C Wang Y Duan H Pan M Zhao J . Efficacy and safety of neoadjuvant immunotherapy in surgically resectable esophageal cancer: A systematic review and meta-analysis. Int J Surg. (2022) 104:106767. doi: 10.1016/j.ijsu.2022.106767, PMID: 35840049

[19] Wang H Zhang X Zhao X Song C Deng W Shen W . Minimal residual disease guided radical chemoradiotherapy combined with immunotherapy after neoadjuvant immunochemotherapy followed by adjuvant immunotherapy for esophageal squamous cell cancer (ECMRD-001): a study protocol for a prospective cohort study. Front Immunol. (2023) 14:1330928. doi: 10.3389/fimmu.2023.1330928, PMID: 38274807 PMC10808458

[20] Wang H Song C Zhao X Deng W Dong J Shen W . Evaluation of neoadjuvant immunotherapy and traditional neoadjuvant therapy for resectable esophageal cancer: a systematic review and single-arm and network meta-analysis. Front Immunol. (2023) 14:1170569. doi: 10.3389/fimmu.2023.1170569, PMID: 37251393 PMC10213267

[21] Raja S Rice TW Lu M Semple ME Blackstone EH Murthy SC . Adjuvant therapy after neoadjuvant therapy for esophageal cancer: who needs it? Ann Surg. (2023) 278:e240–e9. doi: 10.1097/SLA.0000000000005679, PMID: 35997269 PMC10955553

[22] Ni W Yu S Xiao Z Zhou Z Chen D Feng Q . Postoperative adjuvant therapy versus surgery alone for stage IIB-III esophageal squamous cell carcinoma: A phase III randomized controlled trial. Oncologist. (2021) 26:e2151–e60. doi: 10.1002/onco.13914, PMID: 34309117 PMC8649038

[23] Matsuura N Yamasaki M Yamashita K Tanaka K Makino T Saito T . The role of adjuvant chemotherapy in esophageal cancer patients after neoadjuvant chemotherapy plus surgery. Esophagus. (2021) 18:559–65. doi: 10.1007/s10388-020-00811-z, PMID: 33580452

[24] Lordick F Mauer ME Stocker G Cella CA Ben-aharon I Piessen G . Adjuvant immunotherapy in patients with resected gastric and oesophagogastric junction cancer following preoperative chemotherapy with high risk for recurrence (ypN+ and/or R1): European Organisation of Research and Treatment of Cancer (EORTC) 1707 VESTIGE study. Ann Oncol. (2025) 36:197–207. doi: 10.1016/j.annonc.2024.10.829, PMID: 39542422

[25] Liu Z Wang G Yang Y Su Y Zhang H Liu J . ctDNA detects residual disease after neoadjuvant chemoradiotherapy and guides adjuvant therapy in esophageal squamous cell carcinoma. Cell Rep Med. (2025) 6:102334. doi: 10.1016/j.xcrm.2025.102334, PMID: 40914168 PMC12490249

[26] Liu Y Bao Y Yang X Sun S Yuan M Ma Z . Efficacy and safety of neoadjuvant immunotherapy combined with chemoradiotherapy or chemotherapy in esophageal cancer: A systematic review and meta-analysis. Front Immunol. (2023) 14:1117448. doi: 10.3389/fimmu.2023.1117448, PMID: 36761760 PMC9902949

[27] Lin Y Liang HW Liu Y Pan XB . Nivolumab adjuvant therapy for esophageal cancer: a review based on subgroup analysis of CheckMate 577 trial. Front Immunol. (2023) 14:1264912. doi: 10.3389/fimmu.2023.1264912, PMID: 37860010 PMC10582756

[28] Li J Qiu R Hu Y Wang Y Qi Z He M . Postoperative adjuvant therapy for patients with pN+ Esophageal squamous cell carcinoma. BioMed Res Int. (2021) 2021:8571438. doi: 10.1155/2021/8571438, PMID: 33553432 PMC7847342

[29] Lee Y Samarasinghe Y Lee MH Thiru L Shargall Y Finley C . Role of adjuvant therapy in esophageal cancer patients after neoadjuvant therapy and esophagectomy: A systematic review and meta-analysis. Ann Surg. (2022) 275:91–8. doi: 10.1097/SLA.0000000000005227, PMID: 34596073

[30] Zhu Y Sun Y Hu S Jiang Y Yue J Xue X . Comparison of five tumor regression grading systems for gastric adenocarcinoma after neoadjuvant chemotherapy: a retrospective study of 192 cases from National Cancer Center in China. BMC Gastroenterol. (2017) 17:41. doi: 10.1186/s12876-017-0598-5, PMID: 28292272 PMC5351213

[31] Liu BW Liang WD Gu YM Zhang HL Shang QX Chen LQ . Prognostic value of a novel staging system integrating lymph node station number and tumor regression grade for esophageal cancer following neoadjuvant chemoradiotherapy. Ann Surg Oncol. (2026) 33:996–1006. doi: 10.1245/s10434-025-18612-y, PMID: 41207948 PMC12765742

[32] Lin L Yang Y Song X Yu W Li H Zhao L . A pathologic classification approach in esophageal squamous cell carcinoma following neoadjuvant immunochemotherapy: Distinguishing postoperative outcomes and exploring the potential benefit of adjuvant therapy. J Thorac Cardiovasc Surg. (2025) 170:1284–94.e12. doi: 10.1016/j.jtcvs.2025.06.012, PMID: 40543899

[33] Jia W Li C Liu C Hu R . Survival benefit of adjuvant therapy following neoadjuvant therapy in patients with resected esophageal cancer: A retrospective cohort study. PloS One. (2024) 19:e0304937. doi: 10.1371/journal.pone.0304937, PMID: 39561158 PMC11575812

[34] Faiz Z Kats-ugurlu G Mui VEM Karrenbeld A Burgerhof HGM Plukker JTM . Locoregional residual esophageal cancer after neo-adjuvant chemoradiotherapy and surgery regarding anatomic site and radiation target fields: A histopathologic evaluation study. Ann Surg. (2022) 275:e759–e65. doi: 10.1097/SLA.0000000000004242, PMID: 32740246

[35] He W Wang C Li C Nie X Li H Li J . The efficacy and safety of neoadjuvant immunotherapy in resectable locally advanced esophageal squamous cell carcinoma: A systematic review and meta-analysis. Front Immunol. (2023) 14:1118902. doi: 10.3389/fimmu.2023.1118902, PMID: 36875107 PMC9981949

[36] Feng J Wang L Yang X Chen Q . Adjuvant immunotherapy after neoadjuvant immunochemotherapy and esophagectomy for esophageal squamous cell carcinoma: a real-world study. Front Immunol. (2024) 15:1456193. doi: 10.3389/fimmu.2024.1456193, PMID: 39742260 PMC11685212

[37] Abdelhakeem A Blum Murphy M . Adjuvant therapies for esophageal cancer. Thorac Surg Clin. (2022) 32:457–65. doi: 10.1016/j.thorsurg.2022.06.004, PMID: 36266033

[38] Chen YY Wang PP Hu Y Yuan Y Yang YS Shi HS . Clinical efficacy and immune response of neoadjuvant camrelizumab plus chemotherapy in resectable locally advanced oesophageal squamous cell carcinoma: a phase 2 trial. Br J Cancer. (2024) 131:1126–36. doi: 10.1038/s41416-024-02805-5, PMID: 39164491 PMC11442672

