# Supplementary material for: A pathological classification for predicting recurrence and guiding adjuvant therapy in esophageal squamous cell carcinoma following neoadjuvant immunochemotherapy: a two-center cohort study
Source: Front Oncol. 2026 Mar 13;16:1778731. doi: 10.3389/fonc.2026.1778731 (PMC13021421; doi:10.3389/fonc.2026.1778731)
Supplement: Supplementary file 1 [file Table1.doc]

| **Characteristic** | **After PSM** | |  | | **Before PSM (overall population)** | | | |  |
| --- | --- | --- | --- | --- | --- | --- | --- | --- | --- |
|  | **Nonadjuvant (N=68)** | **Adjuvant**  **(N=68)** | | ***P*** | | **Nonadjuvant**  **(N=103)** | **Adjuvant**  **(N=180)** | ***P*** | |
| **Age, y, mean±SD** | 66.8±7.0 | 66.9±7.3 | | 0.935 | | 68.0±7.3 | 66.8±7.0 | 0.173 | |
| **ypT 0** | 31 | 27 | | 0.779 | | 45 | 46 | 0.006 | |
| **ypT 1** | 4 | 6 | |  |  | 11 | 14 |  |  |
| **ypT 2** | 10 | 13 | |  |  | 14 | 30 |  |  |
| **ypT 3** | 23 | 22 | |  |  | 33 | 84 |  |  |
| **ypT 4a** | 0 | 0 | |  |  | 0 | 6 |  |  |
| **ypN 0** | 37 | 39 | | 0.558 | | 67 | 66 | < 0.001 | |
| **ypN 1** | 24 | 21 | |  |  | 29 | 67 |  |  |
| **ypN 2** | 4 | 7 | |  |  | 4 | 36 |  |  |
| **ypN 3** | 3 | 1 | |  |  | 3 | 11 |  |  |
| **TRG 0** | 21 | 21 | | 0.692 | | 34 | 30 | < 0.001 | |
| **TRG 1** | 26 | 21 | |  |  | 48 | 36 |  |  |
| **TRG 2** | 10 | 10 | |  |  | 10 | 50 |  |  |
| **TRG 3** | 11 | 16 | |  |  | 11 | 64 |  |  |

**TABLE A1. Baseline characteristics by adjuvant therapy: overall and post-PSM**
