# Supplementary material for: A pathological classification for predicting recurrence and guiding adjuvant therapy in esophageal squamous cell carcinoma following neoadjuvant immunochemotherapy: a two-center cohort study
Source: Front Oncol. 2026 Mar 13;16:1778731. doi: 10.3389/fonc.2026.1778731 (PMC13021421; doi:10.3389/fonc.2026.1778731)
Supplement: Supplementary file 2 [file Table2.doc]

**TABLE A2.Univariate cox results**

| **Variable** | **HR** | **CI_lower** | **CI_upper** | ***p_*value** |
| --- | --- | --- | --- | --- |
| Age | 0.996 | 0.969 | 1.023 | 7.52E-01 |
| Weight | 0.973 | 0.953 | 0.993 | 9.50E-03 |
| Height | 0.984 | 0.959 | 1.01 | 2.20E-01 |
| tumor location:Upper thoracic | 1 | *NA* | *NA* | *NA* |
| tumor location:Middle thoracic | 0.972 | 0.493 | 1.914 | 9.34E-01 |
| tumor location:Lower thoracic | 1.319 | 0.662 | 2.625 | 4.31E-01 |
| tumor length | 1.013 | 0.916 | 1.121 | 7.98E-01 |
| ypT0 | 1 | *NA* | *NA* | *NA* |
| ypT1 | 1.987 | 0.81 | 4.876 | 1.34E-01 |
| ypT2 | 5.299 | 2.826 | 9.937 | 2.01E-07 |
| ypT3 | 3.018 | 1.694 | 5.375 | 1.77E-04 |
| ypT4a | 14.338 | 4.672 | 44.002 | 3.25E-06 |
| ypN0 | 1 | *NA* | *NA* | *NA* |
| ypN1 | 3.091 | 1.885 | 5.068 | 7.72E-06 |
| ypN2 | 7.65 | 4.417 | 13.252 | 3.89E-13 |
| ypN3 | 4.577 | 2.057 | 10.182 | 1.93E-04 |
| No.lymph nodes | 1.008 | 0.992 | 1.025 | 3.22E-01 |
| MPR | 0.333 | 0.22 | 0.504 | 1.94E-07 |
| pCR | 0.379 | 0.207 | 0.692 | 1.57E-03 |
| TRG0 | 1 | *NA* | *NA* | *NA* |
| TRG1 | 1.379 | 0.678 | 2.802 | 3.75E-01 |
| TRG2 | 2.96 | 1.499 | 5.848 | 1.78E-03 |
| TRG3 | 4.232 | 2.233 | 8.02 | 9.75E-06 |
| Nonadjuvant therapy | 1 | *NA* | *NA* | *NA* |
| Adjuvant therapy:paic | 1.853 | 1.201 | 2.857 | 5.26E-03 |
| Adjuvant therapy:pai | 0.929 | 0.477 | 1.812 | 8.30E-01 |
| nerve invasion | 2.597 | 1.759 | 3.834 | 1.57E-06 |
| vascular thrombosis | 2.967 | 2.014 | 4.371 | 3.73E-08 |
| pdl1_expression | 1.542 | 1.029 | 2.31 | 3.57e-02 |
| Ki67:<30% | 1 | *NA* | *NA* | *NA* |
| Ki67:30%-60% | 1.941 | 1.191 | 3.164 | 7.79E-03 |
| Ki67:>60% | 2.093 | 1.228 | 3.568 | 6.64E-03 |
