# Supplementary material for: A pathological classification for predicting recurrence and guiding adjuvant therapy in esophageal squamous cell carcinoma following neoadjuvant immunochemotherapy: a two-center cohort study
Source: Front Oncol. 2026 Mar 13;16:1778731. doi: 10.3389/fonc.2026.1778731 (PMC13021421; doi:10.3389/fonc.2026.1778731)
Supplement: Supplementary file 3 [file Table3.doc]

**TABLE A3.Multivariate cox results**

| Variable | **HR** | **CI_lower** | **CI_upper** | ***p*_value** |
| --- | --- | --- | --- | --- |
| ypN1 | 4.741 | 2.758 | 8.148 | 0 |
| ypN2 | 5.641 | 2.801 | 11.36 | 0 |
| ypN3 | 3.49 | 1.386 | 8.79 | 0.008 |
| vascular thrombosis | 0.926 | 0.556 | 1.543 | 0.7677 |
| MPR | 2.086 | 0.461 | 9.443 | 0.3401 |
| ypT1 | 3.81 | 0.93 | 15.605 | 0.063 |
| ypT2 | 7.888 | 2.172 | 28.652 | 0.0017 |
| ypT3 | 3.516 | 0.925 | 13.371 | 0.065 |
| ypT4 | 23.669 | 4.43 | 126.467 | 0.0002 |
| nerve invasion | 2.351 | 1.445 | 3.825 | 0.0006 |
| TRG1 | 0.293 | 0.078 | 1.095 | 0.0679 |
| TRG2 | 0.862 | 0.504 | 1.475 | 0.5887 |
| TRG3 | *NA* | *NA* | *NA* | *NA* |
| pCR | *NA* | *NA* | *NA* | *NA* |
| Adjuvant therapy:paci | 0.765 | 0.439 | 1.333 | 0.3441 |
| Adjuvant therapy:pai | 0.705 | 0.338 | 1.472 | 0.3521 |
| Ki67:30%-60% | 1.584 | 0.901 | 2.786 | 0.1104 |
| Ki67:>60% | 1.536 | 0.838 | 2.817 | 0.165 |
| Weight | 0.982 | 0.962 | 1.002 | 0.0816 |
| pdl1 expression≥1% | 0.846 | 0.52 | 1.376 | 0.4993 |
