# Supplementary figures and images for: A pathological classification for predicting recurrence and guiding adjuvant therapy in esophageal squamous cell carcinoma following neoadjuvant immunochemotherapy: a two-center cohort study
Source: Front Oncol. 2026 Mar 13;16:1778731. doi: 10.3389/fonc.2026.1778731 (PMC13021421; doi:10.3389/fonc.2026.1778731)

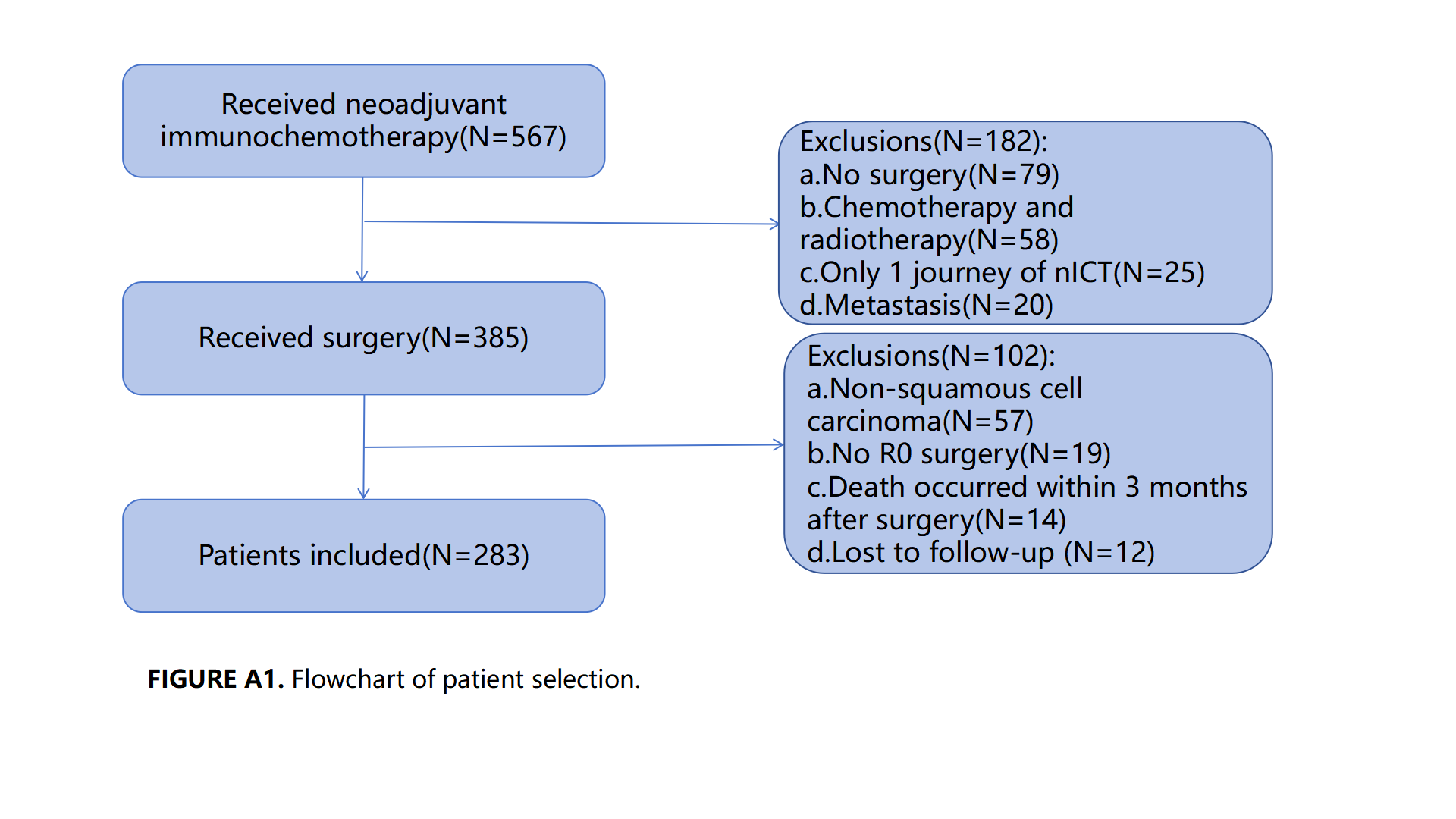

Supplement: Supplementary file 4 [file Image1.tif]

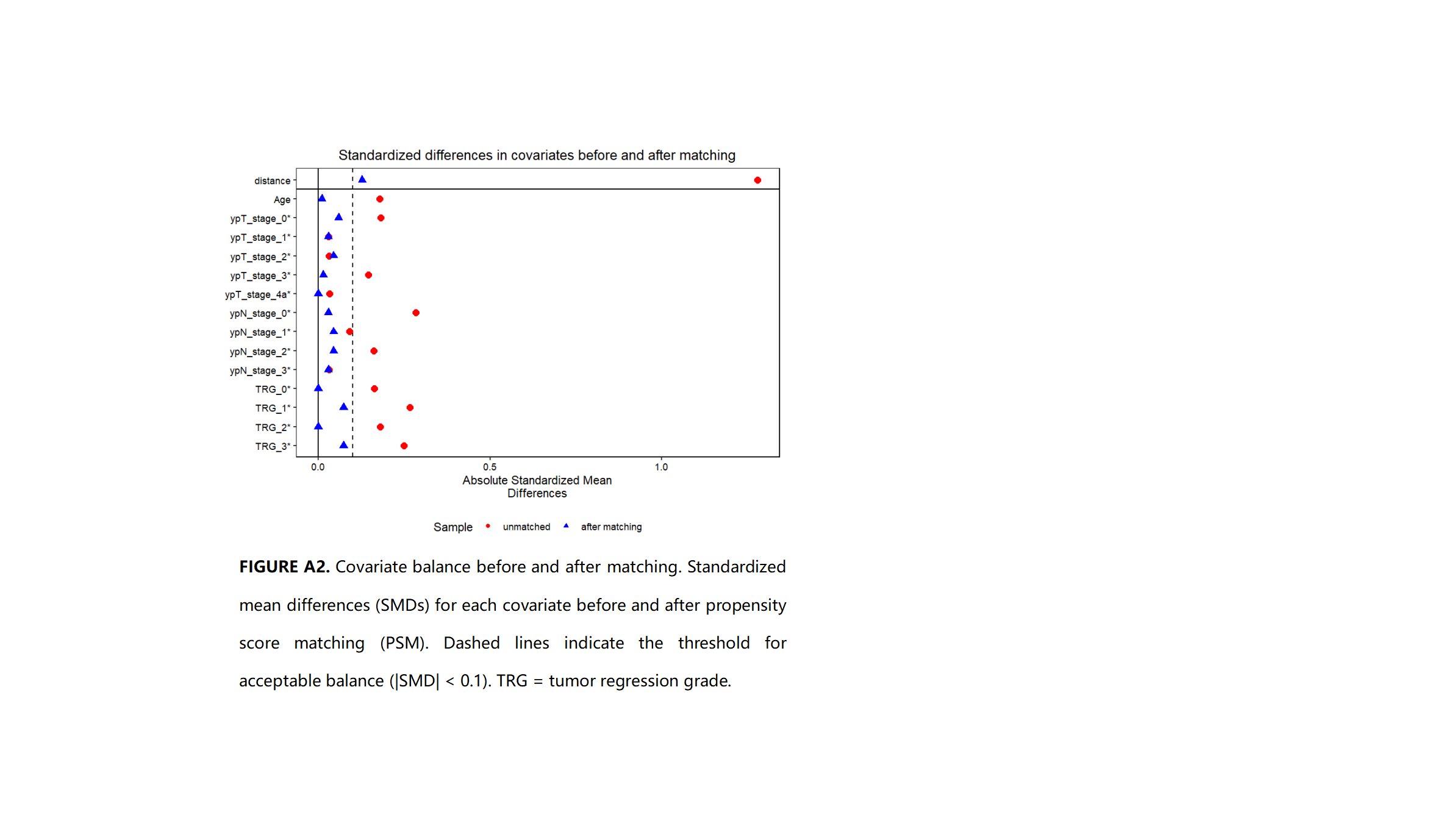

Supplement: Supplementary file 5 [file Image2.tif]

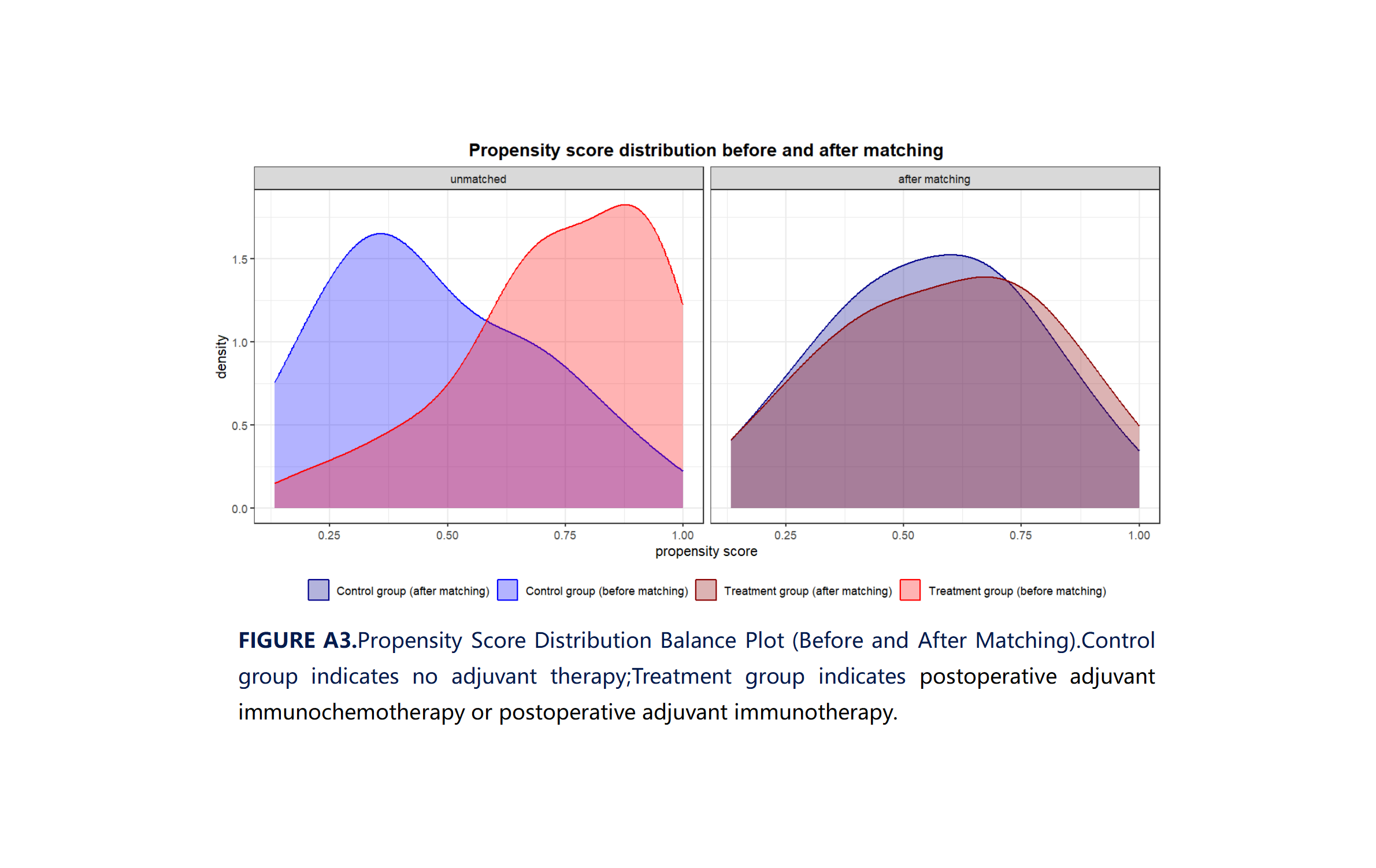

Supplement: Supplementary file 6 [file Image3.tif]

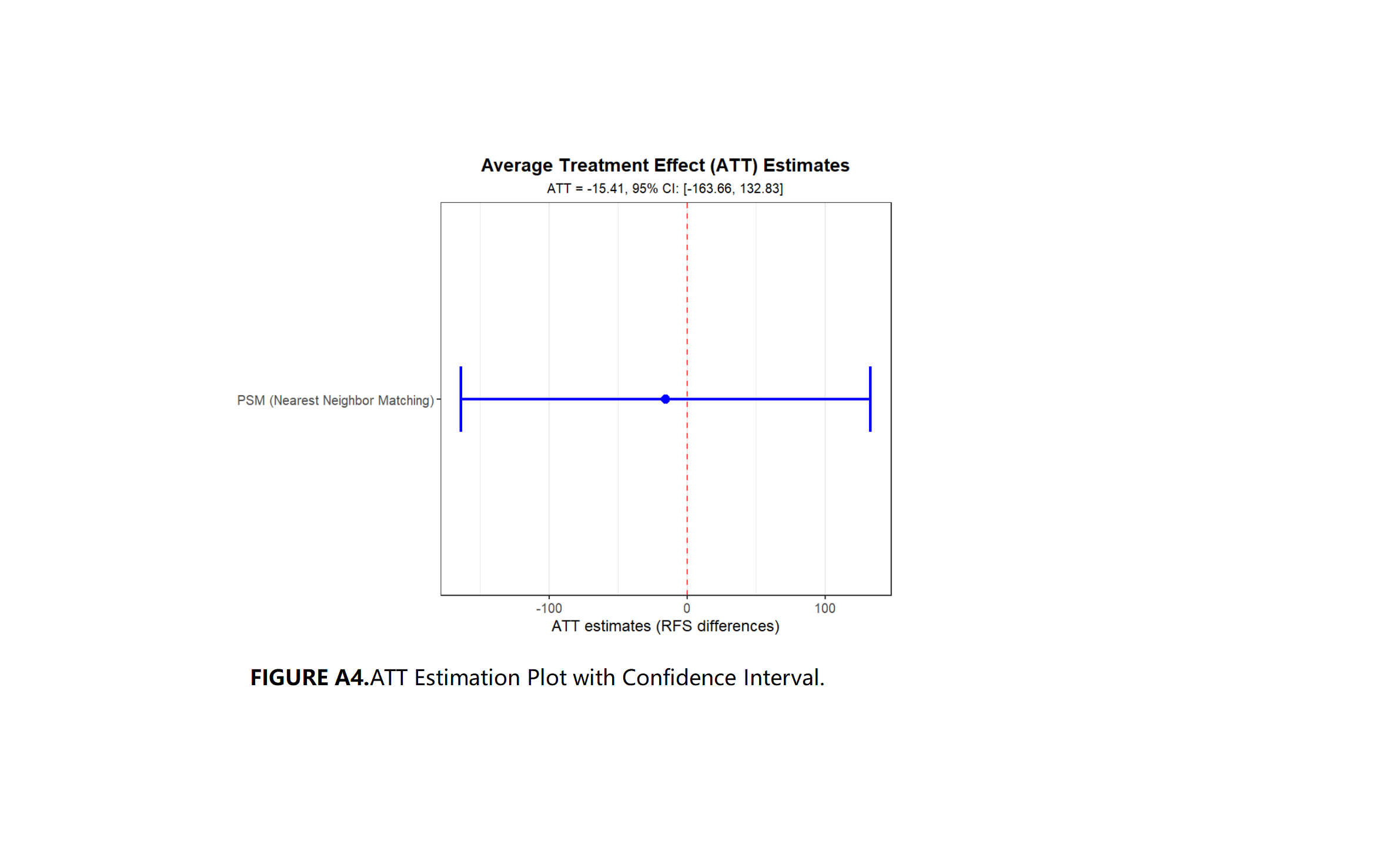

Supplement: Supplementary file 7 [file Image4.tif]

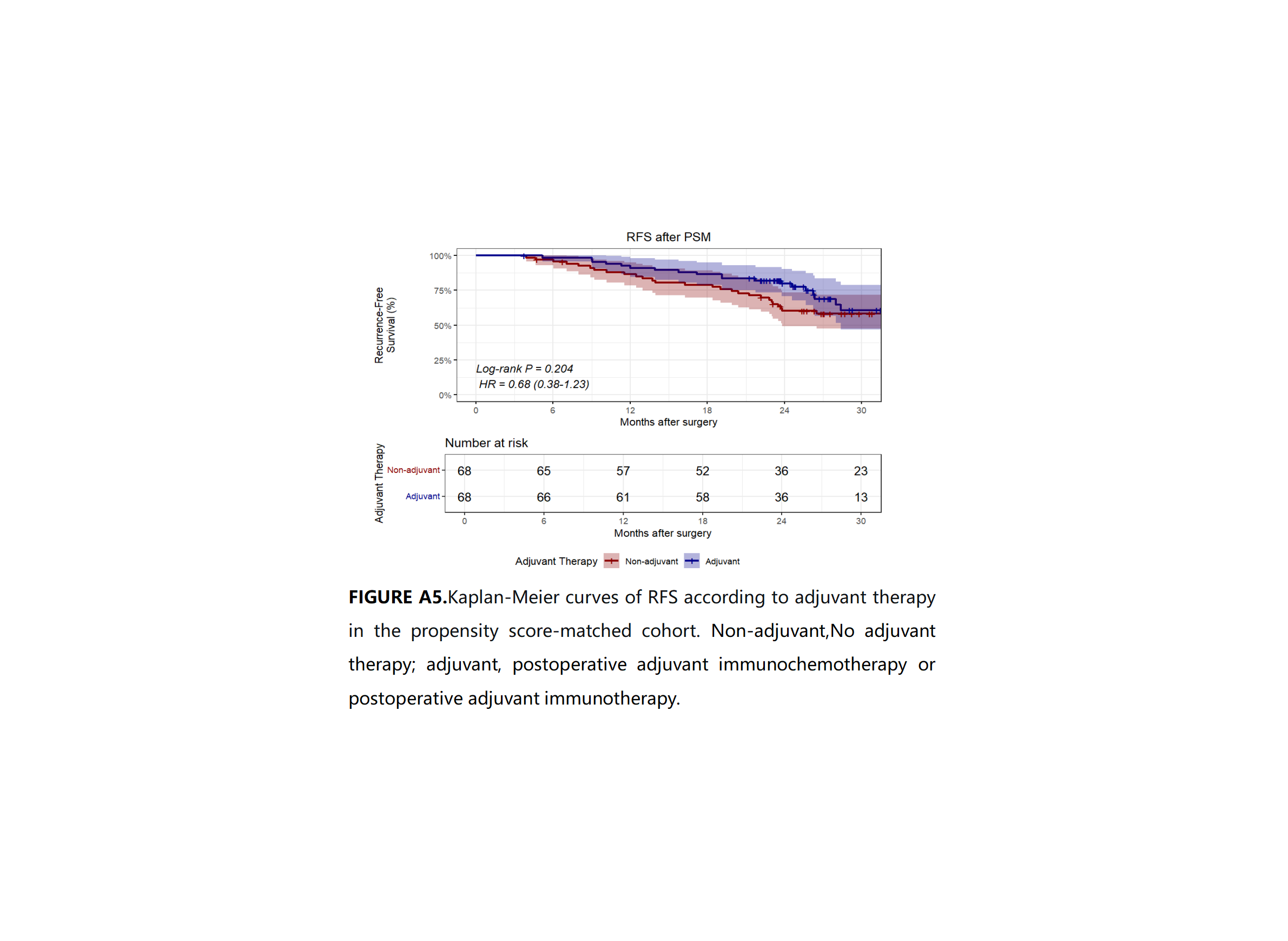

Supplement: Supplementary file 8 [file Image5.tif]

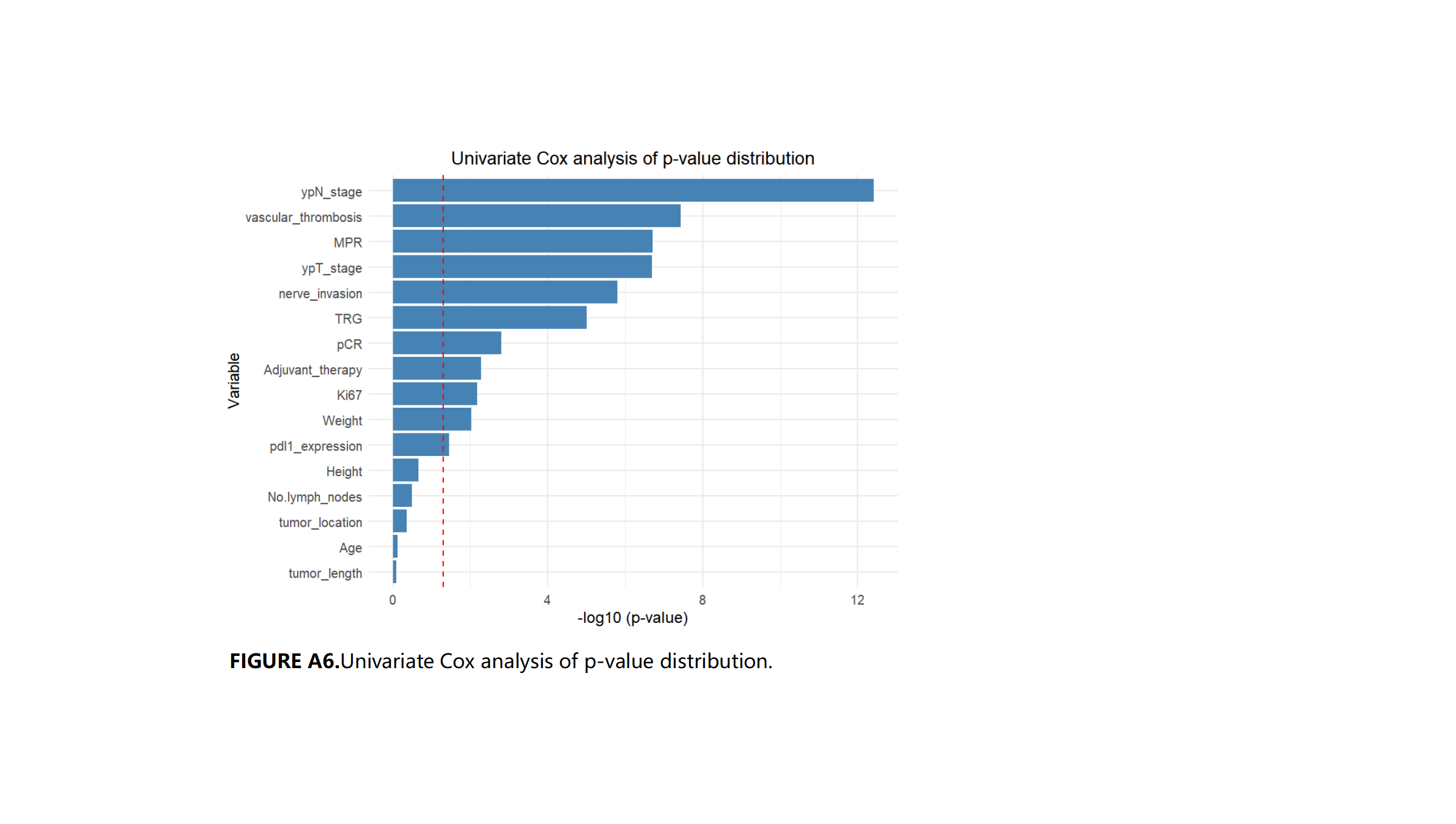

Supplement: Supplementary file 9 [file Image6.tif]
